# Incorporation of a TGF-β2-inhibiting oligodeoxynucleotide molecular adjuvant into a tumor cell lysate vaccine to enhance antiglioma immunity in mice

**DOI:** 10.3389/fimmu.2023.1013342

**Published:** 2023-01-27

**Authors:** Liqun Tu, Zhe Wang, Lei Yang, Xiaomeng Sun, Yunpeng Yao, Peng Zhang, Xiaotian Zhang, Liying Wang, Yongli Yu, Ming Yang

**Affiliations:** ^1^ Department of Immunology, College of Basic Medical Sciences, Jilin University, Changchun, Jilin, China; ^2^ Department of Molecular Biology, College of Basic Medical Sciences, Jilin University, Changchun, Jilin, China; ^3^ Department of Thoracic Surgery, The First Hospital of Jilin University, Changchun, Jilin, China; ^4^ Department of Clinical Laboratory, The Second Hospital of Jilin University, Changchun, Jilin, China

**Keywords:** adjuvant, tumor vaccines, TGF-β2, inhibitory oligodeoxynucleotide, glioma

## Abstract

**Introduction:**

Transforming growth factor β2 (TGF-β2), also known as glioma-derived T-cell suppressor factor, is associated with the impairment of tumor immune surveillance. Therefore, blocking TGF-β2 signaling probably be a feasible strategy to develop a novel type of adjuvant for glioma vaccines to enhance antitumor immunity.

**Methods:**

A TGF-β2 inhibitory oligodeoxynucleotide, TIO3, was designed with sequences complementary to the 3' untranslated region of TGF-β2 mRNA. The expression of TGF-β2 and MHC-I was detected by qPCR, western and flow cytometry in vitro. All the percentage and activation of immune cells were detected by flow cytometry. Subsequently, TIO3 was formulated with Glioma cell lysate (TCL) and investigated for its antitumor effects in GL261 murine glioma prophylactic and therapeutic models.

**Results:**

TIO3 could efficiently downregulate the expression of TGF-β2 while increase the MHC-I's expression in GL261 and U251 glioma cells in vitro. Meanwhile, TIO3 was detected in mice CD4+ T, CD8+ T, B and Ly6G+ cells from lymph nodes after 24 hours incubation. Moreover, TCL+TIO3 vaccination significantly prolonged the survival of primary glioma-bearing mice and protected these mice from glioma re-challenge in vivo. Mechanistically, TCL+TIO3 formulation strongly evoke the antitumor immune responses. 1) TCL+TIO3 significantly increased the composition of CD4+ and CD8+ T cells from draining lymph nodes while promoted their IFN-γ production and reduced the expression of TGF-β2 and PD1. 2) TCL+TIO3 activated the NK cells with the elevation of CD69 or NKG2D expression and PD1 reduction. 3) TCL+TIO3 increased the glioma-specific lysis CTLs from spleen. 4) TCL+TIO3 downregulated PD-L1 expression in glioma tissues and in Ly6G+ cells among glioma-infiltrating immune cells.

**Conclusion:**

TIO3 is a promising adjuvant for enhancing TCL-based vaccines to produce a more vigorous and long-lasting antitumor response by interfering with TGF-β2 expression.

## 1 Introduction

Glioma is a frequent type of brain tumor that is characterized by a poor prognosis and high mortality. Currently, the most common therapeutic treatments include surgery, radiation, and chemotherapy; however, the median survival of patients with glioma is just 15 months, emphasizing the need for novel therapeutic approaches (1). With the development of cancer treatments, tumor immunotherapy has become an innovative maintenance approach for the treatment of glioma. Immunotherapy can effectively promote immune effector cells to infiltrate the brain and specifically destroy residual tumor cells with no collateral damage in critical neural tissues, as confirmed in many preclinical studies (2).

Tumor vaccines are a promising form of immunotherapy that can activate the immune system to combat tumor growth. Over the last two decades, tumor vaccines have attracted increasing attention for their application in tumor prevention and treatments. Remarkably, Provenge, as the first tumor vaccine for the treatment of advanced prostate cancer, was approved by the U.S. Food and Drug Administration (FDA) in April 2010 (3). Moreover, numerous strategies have been developed to generate prophylactic or immunotherapeutic tumor vaccines with encouraging antitumor immune responses that have been undergoing clinical trials in cancer patients (4). Tumor vaccines based on single or combined tumor-associated antigens (TAAs) are a common formulation and preparation strategy (5). However, these vaccines are restricted to the subset of patients whose tumors express the known TAAs, and increasing immune responses may ultimately have limited efficacy due to tumor heterogeneity and loss of antigen expression over time (6). Compared with single tumor antigen strategies, tumor cell lysate (TCL) as the source of various antigens offers the potential advantage of inducing a broad T-cell response against multiple known and unknown TAAs expressed by the specific tumor, which facilitates a reduction in tumor escape (7). In a clinical research trial, dendritic cells (DCs) loaded with glioma TCL displayed a more potent antitumor effect than glioma-associated antigen-loaded DCs (8). Although glioma lysates offer a large variety of antigenic peptides, the immunogenicity of each antigen can vary widely depending on how efficiently it is bound to major histocompatibility complex (MHC) molecules and presented by DCs, resulting in suboptimal or inconsistent immune responses against tumors (9). Therefore, glioma TCL needs to be prepared into a formulation with effective adjuvants to enhance the immune responses against glioma.

Accumulated evidence has revealed that immune-stimulating cytokines or immunosuppressive cytokine inhibitors are considered potent adjuvants in tumor cell vaccines (10). Transforming growth factor-beta 2 (TGF-β2) is a cytokine that plays a very important role in tumor initiation, progression, and many other important processes in malignancy. Generally, TGF-β2 has been identified to negatively modulate the expression of MHC molecules to downregulate both innate and adaptive immune responses. Several studies found that TGF-β2 can inhibit the expression of MHC I, MHC II and CD80, CD86 and CD40 molecules on macrophages and dendritic cells and partially block the rat astrocyte autoantigen presentation and upregulation of MHC I and MHC II induced by interferon-γ (IFN-γ) (11, 12). Additionally, TGF-β2 can induce the differentiation of naive CD4^+^ T cells into conventional CD4^+^ CD25^+^ Foxp3^+^ regulatory T cells (Tregs) by binding to TβRIII (13). Therefore, inhibition of TGF-β2 may promote antigen-presenting cells (APCs) to express MHC molecules/costimulatory molecules and reduce the generation of Tregs to enhance immune responses.

TGF-β2 inhibitors have been used in cancer therapy studies. Short hairpin RNAs (shRNAs) and antisense transgenes have been used extensively in mediating the knockdown of TGF-β2 expression in cells. An oncolytic adenovirus expressing a TGF-β2 shRNA and granulocyte-macrophage colony stimulating factor (GM-CSF) can significantly enhance the anti-melanoma efficacy of melanoma antigen Melan-A (MART1) in mice (14). Similarly, the coexpression of GM-CSF and TGF-β2 antisense transgenes in an irradiated autologous whole-cell vaccine was used in a phase I/II clinical trial for treating patients with advanced prostate cancer, colon carcinoma, gastric cancer and leiomyosarcoma (15). Moreover, a therapeutic vaccine known as Belagenpumatucel-L, which is comprised of four TGF-β2 interfering gene-modified, irradiated, allogeneic non-small cell lung cancer (NSCLC) cell lines, has also been evaluated in a phase III clinical trial (16). Together, these data implied that TGF-β2 inhibitors could facilitate the stimulation of immune responses against TAAs and break immune tolerance, eliciting more vigorous antitumor responses. As a promising TGF-β2 inhibitor, specific oligodeoxynucleotides (ODNs) targeting TGF-β2 have also been developed to directly treat some types of cancer, such as high recurrence of malignant glioma, pancreatic cancer, melanoma, and triple-negative breast cancer (17–20). However, it is still uncertain whether TGF-β2 antisense oligodeoxynucleotides (ASOs) can act as effective tumor vaccine adjuvants, and the specific mechanism by which TGF-β2 ASOs enhance antitumor immune responses is still elusive due to limited research findings.

In a previous study, we designed a TGF-β2 inhibitory oligodeoxynucleotide (TIO3) with sequences complementary to the 3’ UTRs of TGF-β2 mRNA and demonstrated that TIO3 could enhance multiple microbial vaccines to induce strong and persistent antibody responses by suppressing TGF-β2 expression in immune cells (12). In the current study, TIO3 was used as an adjuvant in a formulation with the glioma TCL, which was used to investigate the antitumor effects of TGF-β2 inhibition in mouse GL261 glioma models. Our results indicated that TIO3-TCL regulates the activation of potent glioma-specific cytotoxic T lymphocytes and prolongs the survival of mice with *in situ* glioma. Furthermore, TIO3 activated T and NK cells in the lymph nodes and inhibited PD1 and PD-L1 expression in mice bearing GL261 glioma. These data support the impact of TIO3 on antitumor immunity, allowing the advancement of TIO3 as a promising adjuvant for glioma vaccines.

## 2 Materials and methods

### 2.1 Oligodeoxynucleotides

Two ODNs fully phosphonothioate-modified were used in our study: TIO3, 5’-TTACCACTAGAGCACCACA-3’ (19 nt); control ODN (cODN), 5’-ACTTACTCGAGAACCCCAA-3’ (19 nt). All ODNs, including 3’ Alexa Fluor 488-conjugated TIO3 and 3’ Cy3-conjugated TIO3, were synthesized by Sangon Biotech (Shanghai, China).

### 2.2 Cell lines

GL261 cells (a murine glioma cell line) and U251 cells (a human glioblastoma cell line) were kindly provided by the Transfusion Research Institute of the Academy of Military Sciences, Beijing, China. Cells were maintained in RPMI-1640 or DMEM (Gibco, Carlsbad, CA, USA) supplemented with 10% heat-inactivated fetal bovine serum (Biological Industries, TBD, Tianjin, China), 100 U/mL penicillin and 100 mg/mL streptomycin in a humidified 5% CO_2_ incubator at 37°C. In this study, GL261 cells were used for *in vitro* cell culture experiments and *in vivo* tumor challenge experiments. For *in vivo* tumor challenge experiments, GL261 cells were digested with trypsin-EDTA (0.25%), harvested, centrifuged for 5 min at 300 g, resuspended and then adjusted to a concentration of 5 × 10^6^ cells/mL in serum-free RPMI-1640 for inoculating the mice with 2× 10^4^ cells through an intracranial injection.

### 2.3 Mice

Six-week-old female C57BL/6 mice were purchased from Vital River Laboratory Animal Technology Co., Ltd. (Beijing, China). Experimental procedures and manipulation involving mice were performed in accordance with the National Institute of Health Guide for the Care and Use of Laboratory Animals and approved by the Scientific Investigation Board of Science & Technology of Jilin Province and the ethics committee of the College of Basic Medical Sciences of Jilin University (number 2019-01).

### 2.4 Preparation of GL261 tumor cell lysate

The tumor cell lysates were prepared as described previously (21). Briefly, GL261 cells were cultured and harvested *in vitro*, then 10^6^ cells were intraperitoneally inoculated into the mice. Glioma mass was formed in about 20 days, then the mice was euthanized to isolate glioma tissue for preparing glioma cell lysate. To acquire high-quality glioma cell lysates, we first minced the glioma tissues with scissors, resuspended in 0.85% pathogen-free saline buffer and then disrupted in a glass homogenizer followed by filtration with a 40 μm nylon cell strainer. Cells were adjusted with the concentration at 10^7^ cells/mL in saline and then disrupted by high pressure homogenizer at 800 Bar 5 cycles. After centrifugation at 10,000 g for 10 mins, the cell supernatant was separated and passed through a 0.2 μm filter. At last, the tumor cell lysate was conducted freeze-dried for storage at -80 °C (21). TCLs were detected under a microscope (Olympus Corporation, Tokyo, Japan) using trypan blue staining (Sigma-Aldrich, St. Louis, MO, USA) (22).

### 2.5 RNA isolation and reverse transcription

GL261 and U251 cells were prepared for isolating total RNA with TRIzol™ Reagent (Invitrogen, Carlsbad, CA). A UV2800 ultraviolet spectrophotometer was used to analyze the RNA concentration and purity. M-MLV reverse transcriptase (part: 28025021; Invitrogen, UK) was used for reverse transcription reactions. Briefly, the RT reaction mixture, in a volume of 20 µL, contained MgCl_2_ (3 mM), dNTP mix (0.5 μM), 1 μl M-MLV, 1 µg of total RNA, oligo(dT)15 primer (0.5 µg) and reaction buffer. The RT reaction was incubated at 42°C for 1 h, and the RT enzyme was inactivated by incubation at 70°C for 15 min.

### 2.6 Real-time quantitative polymerase chain reaction

To quantify the mRNA expression of TGF-β2 and MHC-I, we used primers synthesized by Sangon Biotech (Shanghai, China), which are listed in **Table 1**, to amplify the target gene. Quantitative real-time polymerase chain reaction (RT−PCR) was performed using two-step SYBR green RT−PCR assays (Transgene Biotech, G31227) in a Step One Real-Time PCR System (Applied Biosystems, Foster City, CA, USA) (12). The procedure for qRT−PCR was one cycle at 95°C (30 s) followed by 40 cycles at 95°C (5 s) and 64°C (31 s) (12). *GAPDH*, the most commonly used gene for normalization of qPCR data in glioma research, was used (23). Relative mRNA expression was calculated after normalizing cycle thresholds against *GAPDH* and is presented as the fold change value (2^ΔΔ^comparative threshold) relative to the control.

**Table 1:** Primers used in real time-PCR.

Primer name	Oligonucleotides sequences (5’-3’)
mTGF-β2-F	TCGACATGGATCAGTTTATGCG
mTGF-β2-R	CCCTGGTACTGTTGTAGATGGA
mMHC-I-F	TACCTGAAGAACGGGAACGC
mMHC-I-R	CCATTCAACTGCCAGGTCAG
mGAPDH-F	ATCACCATCTTCCAGGAGCGA
mGAPDH-R	TCTCGTGGTTCACACCCATCA
hTGF-β2-F	CAGCACACTCGATATGGACCA
hTGF-β2-R	CCTCGGGCTCAGGATAGTCT
hMHC-I-F	CAGATACCTGAAGAACGGGAAC
hMHC-I-R	GCACCTCAGGGTGACTTTAT
hGAPDH-F	GGAGCGAGATCCCTCCAAAAT
hGAPDH-R	GGCTGTTGTCATACTTCTCATGG

### 2.7 Flow cytometry assay

All anti-mouse or anti-human monoclonal antibodies (mAbs) in this study were purchased from BD Biosciences (Franklin Lakes, NJ, USA).

To analyze the surface TGF-β2 (sTGF-β2) and MHC-I expression on GL261 cells, GL261 cells were cultured with PMA (100 μg/mL), PMA+TIO3 (10 μg/mL), or PMA+cODN (10 μg/mL) for 24 h and then stained with an anti-MHC-I mAb (PE-conjugated, 566776) or goat-anti-mouse TGF-β2 antibody (AB-112N, R&D system, USA) for approximately 40 minutes at 4°C in the dark. After being washed twice with FACS buffer (PBS, containing 2 mmol/L EDTA and 20 mL/L FBS), the cells were incubated with the Alexa Fluor^®^488-conjugated donkey-anti-goat IgG secondary antibody (ab150129) diluted with PBS at 1:1000 for 40 min and then analyzed by flow cytometry (FACSCalibur, Becton Dickinson, USA).

To analyze the effect of TIO3 on the expression of TGF-β2 and IFN-γ in CD4^+^ T cells and CD8^+^ T cells, the mice were intramuscularly immunized with TCL, TCL+TIO3, TCL+cODN or PBS at the cervical lymph node area on Day 0 and Day 10. On Day 14, the lymphocytes from drainage lymph nodes were isolated. The cells were first stained with PE-labeled CD4 mAb and goat-anti-mouse TGF-β2 antibody or with PE-labeled CD8 mAb and goat-anti-mouse TGF-β2 antibody for 40 minutes on ice in the dark. After being washed twice, the cells were incubated with Alexa Fluor^®^488-conjugated donkey-anti-goat IgG secondary antibody diluted with PBS at 1:1000 for 40 min. For intracellular staining, cells were fixed in 4% paraformaldehyde and permeabilized in 0.1% saponin before staining with APC-labeled IFN-γ mAb (554413), followed by flow cytometry analysis. Similarly, to investigate the effect of TIO3 on the expression of CD69, NKG2D and PD1 in NK cells and CD4^+^ and CD8^+^ T cells, lymphocytes isolated from immunized mice were first stained with APC-conjugated anti-NK1.1 mAb (561117), PE-conjugated anti-CD4 mAb (553048), PE-conjugated anti-CD8 mAb (553033), PE-conjugated anti-CD314 mAb (NKG2D, 558403), FITC-labeled CD279 (PD1) and/or FITC-conjugated anti-CD69 mAb (553236) for 40 minutes on ice in the dark and then analyzed by BD FACS Calibur with Cell Quest software (BD Bioscience, San Jose, CA).

To explore the effect of TIO3 on glioma-specific T cells in tumor-bearing mice, C57BL/6 mice were intramuscularly immunized with 100 μL of TCL (100 μg TCL in PBS), TCL + TIO3 (100 μg TCL plus 10 μg TIO3 in PBS), TCL+ cODN (100 μg TCL plus 10 μg control ODN in PBS), or PBS on Day 0 and Day 10. On Day 14, the mice were challenged with 2×10^4^ GL261 cells intracranially (i.c.). On Day 28, draining lymph nodes were isolated and minced into a single-cell suspension, and the lymphocytes were stained with PE-labeled anti-CD4 (553048) or PE-labeled anti-CD8 mAb (553033) and FITC-labeled CD69 mAb (553236) at 4°C in the dark for 30 min. After being washed twice, the lymphocytes were analyzed by flow cytometry.

### 2.8 Cytotoxicity assay

The mice were intramuscularly injected with TCL, TCL+TIO3, TCL+cODN or PBS at the cervical lymph node area on Day 0 and Day 10. On Day 14, the spleen was isolated, and then the splenocytes were used to perform the cytotoxicity assay. Splenocytes were cocultured with GL261 (1 × 10^4^ per well per 100 μL) cells at a ratio of 100:1, 50:1 or 25:1 for 8 h at 37 °C in a 5% CO_2_ humidified atmosphere. A methylthiazolyldiphenyl-tetrazolium bromide assay was conducted to determine the CTL activity.

### 2.9 Fluorescence staining and confocal microscopy

To observe whether TIO3 could enter T cells, lymphocytes isolated from naive mice were seeded on poly-L-lysine-coated 24-well plates at a concentration of 2×10^6^ cells/ml in RPMI 1640 supplemented with 10% FBS. The cells were cultured with Cy3-labeled-TIO3 (10 μg/ml) in a 5% CO_2_ incubator for 24 h at 37°C. After washing twice, the cells were fixed with 4% paraformaldehyde for 10 min at 37°C. The cells were washed twice, permeabilized with 0.1% Triton X-100 at 4°C for 10 min, and then blocked with 5% BSA at 37°C for 1 h. The cells were incubated with PE-conjugated CD4 or CD8 mAb at a dilution of 1:50 for 30 min at 4°C. Unbound antibody was washed and removed with PBS. The stained cells were visualized under a laser scanning confocal microscope (Olympus FV1000) and analyzed by AndorIQ2 software.

### 2.10 Effect of TIO3 as a prophylactic and therapeutic glioma vaccine adjuvant in an*in vivo* study

To assess the effect of TIO3 as a prophylactic glioma vaccine adjuvant in brain glioma in situ, 32 female C57 BL/6 mice (n=8 each group) were intramuscularly (i.m.) injected with TCL (100 μg/ml), TCL (100 μg/ml) + TIO3 (10 μg/ml), TCL (100 μg/ml) + cODN (10 μg/ml), or PBS in the neck draining lymph node of the mice on Day 0 and Day 10. On Day 14, each mouse was anesthetized and intracranially (i.c.) injected with 2×10^4^ GL261 cells at 2 mm to the right of the bregma and 3 mm deep using a stereotaxic instrument (Kopf Instruments) (21). Any animals showing clinical symptoms or abnormal neurological signs 1-3 days after surgery were excluded from the experiment. Subsequently, the survival period of the mice after tumor inoculation was recorded.

To explore the effect of TIO3 as a therapeutic glioma vaccine adjuvant in brain glioma in situ, 44 female C57BL/6 mice were split into four groups (n=11), i.c. injected with 2×10^4^ GL261 cells on Day 0 and then i.m. injected with TCL, TCL+TIO3, TCL+cODN or PBS on Days 1, 8, 15 and 22. The mice were monitored daily. In each group, 3 mice were euthanized on Day 18, and the brain tumor tissues were separated and fixed in formalin solution for 24 hours, embedded in paraffin, sectioned (4 μm), and stained with hematoxylin-eosin (H&E) for tumor histopathological analysis under a microscope.

### 2.11 Isolation of tumor-infiltrating immune cells

To isolate the infiltrating immune cells in brain glioma tissues, brain tissue pieces were mechanically processed using collagenase. To digest with collagenase, we first transferred glioma tissue pieces (1 g) to a P60 dish and then incubated them with collagenase IA (5 ml, 100 U/ml in RPMI) at 37°C for 30 minutes. A 5 ml pipette was used to resuspend the sample, which was then transferred into a 15 ml tube and incubated for another 30 minutes at 37°C (24). This step was repeated twice. Thereafter, we filtered the cell suspension by passing it through a 40 µm cell strainer. After centrifugation at 300 g for 10 min at 4 °C, the pellet was resuspended in 4 mL of 30% Percoll (GE) and overlaid on the top of a gradient containing 3.5 mL of 37% and 3.5 mL of 70% Percoll solution (21). Percoll was diluted with Hanks’ balanced salt solution (HBSS) (Bio-Whittaker). The gradient was centrifuged at 500 × g for 20 min at 4 °C; cells were collected from the 37% to 70% interface (approximately 5 mL) and then washed once with HBSS containing 10% fetal bovine serum (FBS). After being washed twice with PBS buffer (containing 20 mL/L FBS), the cells were stained with PE-conjugated CD274 (PD-L1, MIH5) or APC-conjugated Ly6G (560599) mAb for 30 min on ice in the dark. After washing twice, the cells were counted and processed for viability staining and flow cytometric analysis.

### 2.12 Western blotting

To explore the effect of TIO3 on TGF-β2 and PD-L1 expression in glioma tissues, we first i.m. injected the mice with TCL, TCL+TIO3, TCL+cODN, or PBS on Days 0 and Day 10 and then challenged the mice with GL261 cells intracranially on Day 14. On Day 28, the glioma was isolated from the mice and used to assess TGF-β2 and PD-L1 expression by western blotting. The glioma cells were cut into small pieces by scissors first and then digested with collagenase for 1 h. After centrifugation, the cells were lysed with ice-cold radioimmunoprecipitation (RIPA) buffer (150 mM NaCl, 50 mM Tris, pH 7.4, 1% NP-40, 0.5% sodium deoxycholate, 0.1% SDS) containing the protease inhibitor PMSF at 4°C for 30 min. Then, the cell lysates were centrifuged at 14,500 × g for 10 min at 4°C. The cell supernatant was collected and quantified with a BCA protein assay kit (Wanleibio, Shenyang, China) followed by separation on 12% SDS−PAGE. After running the gel at 120 V for 1.5 h, we transferred the protein to polyvinylidene difluoride (PVDF) membranes (Immobilon P; Millipore USA) at 120 V for 1 h at 4°C. Then, the membranes were blocked with TBST containing 5% nonfat dried milk, shaken at room temperature (22-25°C) for 2 h, and probed with an anti-TGF-β2 antibody (AB-112-NA, R&D Systems, USA), anti-PD-L1 antibody (PA5-20343, Invitrogen) or anti-GAPDH antibody (60004-1-Ig, Proteintech) overnight at 4°C. After being washed with TBST [150 mM NaCl, 10 mM Tris-HCl (pH 8.0), 0.5% Tween 20] twice for 10 min intervals, the membranes were incubated with the secondary antibody HRP-conjugated anti-goat IgG (HAF017, R&D Systems, USA) at 25°C for 1 h. After washing three times, substrate was added to the membrane, and the target protein bands were visualized by Hyperfilm ECL (Amersham Biosciences). The intensity of the immunoreactive bands was determined by a densitometric analysis program (Image Gauge V3.12; Fuji Photo Film, Tokyo, Japan).

### 2.13 Statistical analysis

A Student’s 2-tailed unpaired t test was used to determine the statistical significance of differences between two groups. For multiple sets of data analysis, 1- or 2-way ANOVA was used, followed by Scheffé’s *post hoc* test. The Kaplan−Meier method was used to evaluate the survival curve of mice, and the log-rank test was used for comparison. SPSS 19.0 computer software was used for statistical analysis. The data come from four or more independent experimental groups. Data are expressed as the mean ± SEM. Differences in tumor size among the various groups were determined by repeated-measures analysis of variance (ANOVA).

## 3 Results

### 3.1 Regulatory effect of TIO3 on the expression of TGF-β2 and MHC-I in glioma cells

**Figure 1:**
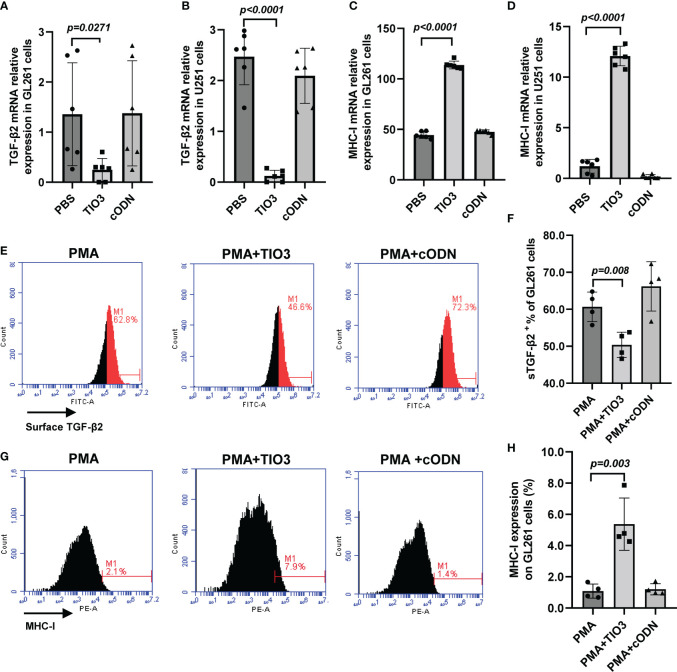
Effect of TIO3 on the expression of TGF-β2 and MHC-I in cultured GL261 cells or U-251 cells. GL261 or U-251 cells were cultured with TCL, TCL+TIO3 (10 μg/mL) or TCL+cODN (control ODN, 10 μg/mL) for 24 h, respectively, and the mRNA expression of TGF-β2 **(A, B)** and MHC I **(C, D)** were measured by qRT-PCR. In addition, GL261 cells were cultured with PMA (100 μg/mL), PMA+TIO3 or PMA+cODN for 48 h, and the protein expression of TGF-β2 **(E, F)** and MHC I **(G, H)** were detected by flow cytometry.

Advanced glioma overproduces TGF-β2, whose autocrine and paracrine actions promote tumor growth, invasion, and metastasis. In this study, we used different types of antigens to induce tumor cells to secrete TGF-β2 and observed the inhibitory effect of TIO3. As expected, TIO3, a specific TGF-β2 inhibitory oligodeoxynucleotide designed in our lab (12), significantly suppressed TGF-β2 mRNA and surface-bound TGF-β2 (sTGF-β2) expression in both GL261 (murine) and U251 (human) glioma cell lines (*p<0.05*) (**Figures 1A, B, E-F**). Moreover, the results also showed that TIO3 significantly upregulated the mRNA and protein expression of MHC I molecules on the surface of GL261 and U251 cells (*p<0.05*) (**Figures 1 C, D, G, H**), which may result in an enhancement of the antitumor immune responses.

### 3.2 Cellular uptake of TIO3 into murine immune cells

**Figure 2:**
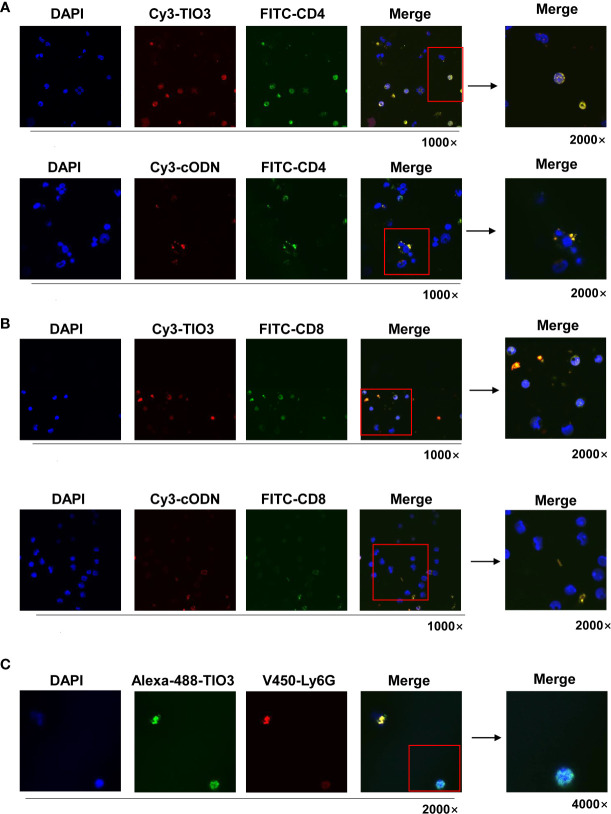
The TIO3 distribution in CD4^+^ T and CD8^+^ T cells. The lymph node (LN) cells from naive mice (n = 4) were incubated with red-fluorescent Cy3-TIO3 or Cy3-cODN or with green-fluorescent Alexa-488-TIO3 for 24 h, then stained with green-fluorescent (FITC-labeled) mAbs against CD4 **(A)** or CD8 **(B)** or red-fluorescent (V450-labeled) mAb against ly6G **(C)**, respectively, followed by counterstaining with DAPI, a blue fluorescent DNA dye for staining the nucleus. The resultant cells were observed under the confocal microscope. (Magnification, × 1000, × 2000, × 4000).

To observe whether TIO3 could be taken up into T cells and neutrophils, murine drainage lymph node (DLN) cells were isolated and incubated with Cy3- or Alexa-488-labeled TIO3 for 24 h and then stained with FITC-labeled mAbs against CD4, CD8, or V450-labeled mAbs against Ly6G, followed by counterstaining with DAPI, a blue-fluorescent DNA dye for staining the nucleus. Confocal microscopy revealed that both TIO3 and cODN could enter CD4^+^ T cells and CD8^+^ T cells, but TIO3 had a higher efficacy than cODN in entering these cells (**Figures 2A, B**). Similarly, TIO3 was also taken up by Ly6G^+^ immune cells (**Figure 2C**), which may result in the inhibition of neutrophil-dependent production of TGF-β2. In addition, TIO3 was confirmed to be ingested by B cells in our previous study (**Figure S1**), facilitating the activation of B cells (12).

### 3.3 Prophylactic protective effect and long-term immune memory of TIO3+TCL in a murine glioma *in situ* model.

**Figure 3:**
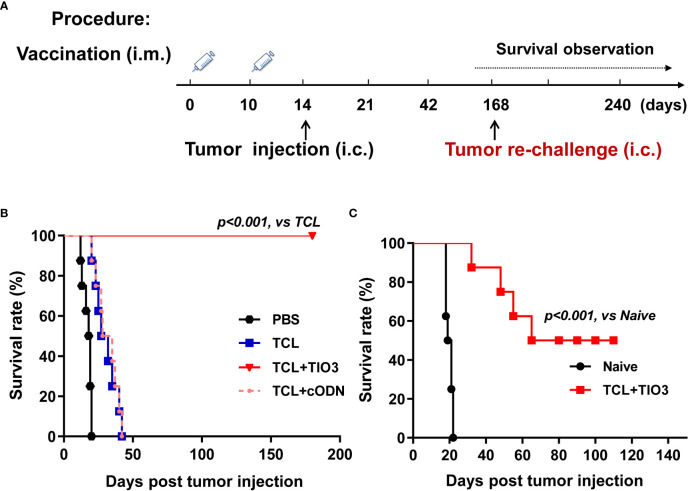
Prophylactic protective effect and anti-tumor immune memory of TIO3 as vaccine adjuvant on the *in-situ* glioma model in mice. **(A)** The experimental procedure: C57BL/6 mice (n=8) were intramuscularly (i.m.) immunized with PBS, TCL, TCL+TIO3 or TCL+cODN twice on day 0 and day 10, and then were challenged intracranially (i.c.) inoculated with 2×10^4^ GL261 cells on day 14. Then, the survived mice were re-challenged with 2×10^4^ GL261 cells on day 168, naive mice (n=8) were intracranially inoculated with 2×10^4^ GL261 cells; **(B)** The survival rate of GL261-bearing mice in different groups after the first tumor inoculation. **(C)** The survival of the mice after tumor re-challenge were observed for another 110 days.

To explore whether TIO3 could be formulated as an adjuvant with glioma TCL to induce antitumor immunity, mice were immunized twice with PBS, TCL, TCL+TIO3 or TCL+cODN (TCL plus control ODN) on Day 0 and Day 10. On Day 14, the mice were intracranially inoculated with GL261 cells. We found that the tumor-bearing mice treated with TCL or PBS exhibited symptoms, including weight loss, hunched back, irregular breathing, prostration, paresis, and convulsions. More excitingly, none of the eight mice in the TCL+TIO3 group died within 180 days (**Figures 3A, B**). The results showed that TIO3 exhibited a potent adjuvant effect and facilitates TCL to induce antitumor immunity against glioma in a prophylactic mouse model.

To observe the immune memory against glioma in TCL+TIO3-immunized mice, the eight surviving mice in the TCL+TIO3 group were rechallenged with intracranial transplantation of GL261 cells on Day 168 **(** **Figure 3A** **)**. Surprisingly, 50% of the mice survived 110 days after glioma rechallenge, reflecting long-lasting immunity against tumors (**Figure 3C**). The data implied that TIO3 could assist TCL in generating tumor-specific immunologic memory.

### 3.4 Effect of TIO3 as a glioma vaccine adjuvant on the activation of CD4^+^ and CD8^+^ T cells

**Figure 4:**
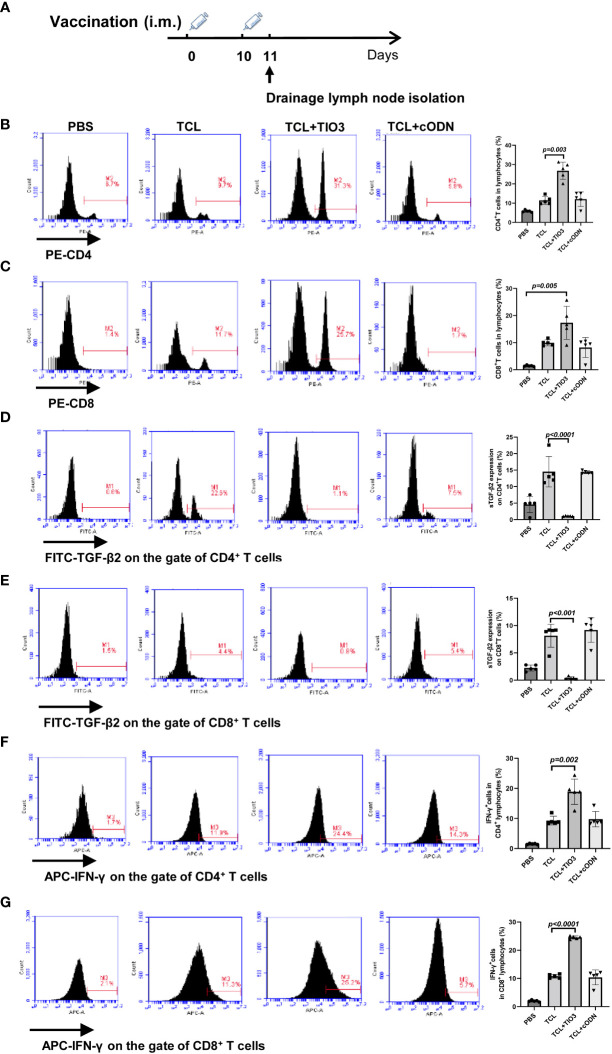
Effect of TIO3 as glioma vaccine adjuvant on the activation of T lymphocyte cells. C57BL/6 mice (n=6) were intramuscularly injected with PBS, TCL, TCL+TIO3, or TCL+cODN on day 0 and day 10, and then the lymphocytes from mouse drainage lymph nodes were isolated and analyzed by FACS. **(A)** Experimental procedure. **(B)** the percentage of CD4^+^ T cells; **(C)** the percentage of CD8^+^ T cells; **(D-G)** the expression percentage of sTGF-β2 and IFN-γ in CD4^+^ T and CD8^+^ T cells.

To explore whether TIO3 as an adjuvant coupled with the TCL vaccine (TCL+TIO3) could induce the generation of effective T cells in mice, C57BL/6 mice were intramuscularly injected with TCL, TCL+TIO3 or TCL+cODN or PBS on Day 0 and Day 10. One day after the second immunization, the lymphocytes were isolated, and the percentage and activation of T cells were immediately determined by flow cytometry (**Figure 4A**). The results showed that TIO3 significantly facilitated the ability of TCL to increase the percentages of CD4^+^ and CD8^+^ T cells in lymphocytes (**Figures 4B, C**). T-cell activation is vital to antitumor immune responses, and therefore the expression of inhibition and activation markers on T cells was observed in this study. As an inhibitory signal of antitumor immunity, the expression of sTGF-β2 on CD4^+^ and CD8^+^ T cells in the TCL+TIO3 group was significantly decreased compared with that in the TCL group (**Figures 4D, E**). IFN-γ secreted by activated T cells can kill tumor cells. As expected, the IFN-γ secretion frequencies of CD4^+^ and CD8^+^ T cells in the TCL+TIO3 group were higher than those in the other groups (**Figures 4F, G**). These results indicated that TIO3 could facilitate TCL’s induction of the activation of T cells.

### 3.5 Effect of TCL+TIO3 on the activation of cytotoxic T lymphocytes and NK cells in glioma-bearing mice

**Figure 5:**
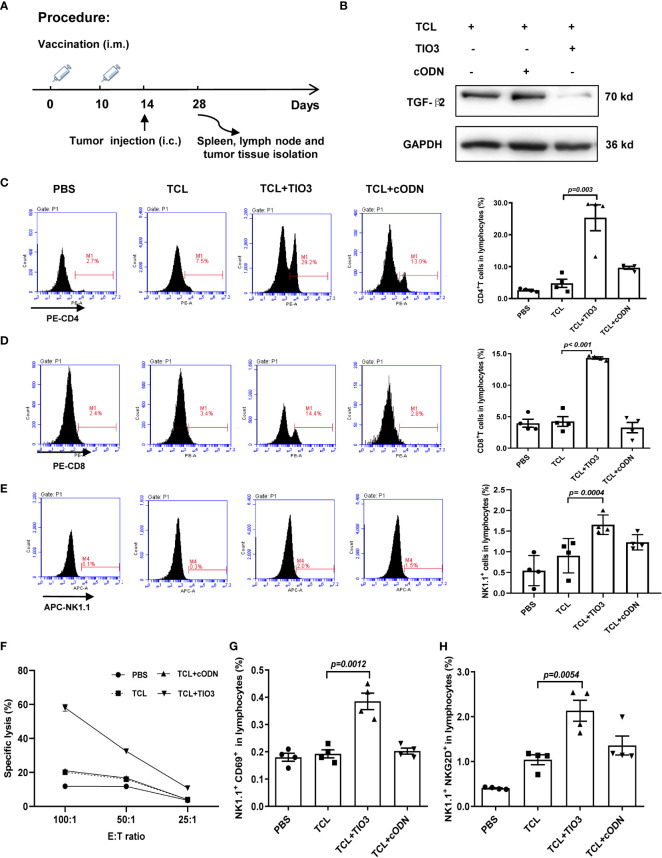
Effect of TCL+TIO3 on the activation of cytotoxic T lymphocytes and NK cell in glioma-bearing mice. The mice (n=4) were intramuscularly immunized with PBS, TCL, TCL+TIO3 and TCL + cODN in the neck on day 0 and 10, and then and then challenged i.c. with 2×10^4^ GL261 cells on day 14. 4 mice were sacrificed on day 28. **(A)** Experimental procedure. **(B)** TGF-β2 expression in glioma tissue isolated from the mice on day 28 by western blotting. **(C-E)** Percentages of CD4^+^ T, CD8^+^ T and NK cells in lymph nodes. **(F)** Glioma-specific CTL detection. Splenocytes were co-cultured with GL261 cells for 8 h at effector/target ratios of 100:1, 50:1 and 25:1, and methylthiazolyldiphenyl-tetrazolium bromide assays was performed for the cytotoxicity. **(G-H)** The expression of CD69 and NKG2D on NK cells in lymphocytes. TIO3 plus TCL *versus* TCL.

To further explore whether TCL+TIO3 also activated specific cytotoxic T lymphocytes (CTLs) in glioma-bearing mice, mice were intramuscularly injected with TCL, TCL+TIO3, TCL+cODN or PBS in the neck on Days 0 and Days 10, and then the mice were i.c. injected with 2×10^4^ GL261 cells in the caudate nucleus. On Day 28, the mice were sacrificed to isolate lymphocytes and analyze T-cell activation, and TGF-β2 expression in glioma tissue was also observed **(** **Figure 5A** **)**. Consistent with the TGF-β2 expression pattern in lymphocytes, western blotting analysis demonstrated that TCL+TIO3 also significantly downregulated TGF-β2 expression in glioma tissue **(** **Figure 5B** **)**. Similarly, our results showed that TCL+TIO3 obviously increased the percentage of CD4^+^ and CD8^+^ T cells in the lymphocytes of glioma-bearing mice (**Figures 5C, D**) compared with the TCL and PBS groups (*p <0.001*). To verify the effect on cytotoxic T cells induced by TCL+TIO3, splenocytes from each group were isolated and cocultured with GL261 cells for 8 h at effector/target ratios of 100:1, 50:1 and 25:1. The results clearly revealed that TCL+TIO3 induced a stronger cytotoxicity against GL261 tumor cells than TCL and PBS *in vitro* (*p <0.001*) (**Figure 5F**), suggesting that TCL+TIO3 could induce significant specific CTLs in glioma-bearing mice. Moreover, we also detected the IFN-γ production of lymphocytes isolated from the mice immunized with TCL+TIO3. The results showed that TCL+TIO3 induced more IFN-γ production than TCL or PBS (**Figure S2**). The IFN-γ production represents that specific CTLs and non-specific NK cell activity.

In addition to T cells, NK cells also play a major role in antitumor immune responses. Thus, the ratio and activation of NK cells in lymphocytes from the immunized mice were also determined by flow cytometry analysis. The results revealed that the ratio of NK cells in the spleen was significantly upregulated by TCL+TIO3 compared with PBS or TCL (*p=0.0004*) (**Figure 5E**). NKG2D and CD69 are activating immune markers expressed by NK cells (25). Our results showed that TCL+TIO3 induced higher ratios of CD69^+^ or NKG2D^+^ NK cells in lymphocytes than TCL (*p<0.01*) (**Figures 5G, H**), indicating the activation of NK cells.

### 3.6 Inhibitory effects of TCL+TIO3 on PD1 or PD-L1 expression in immune cells in immunized mice

**Figure 6:**
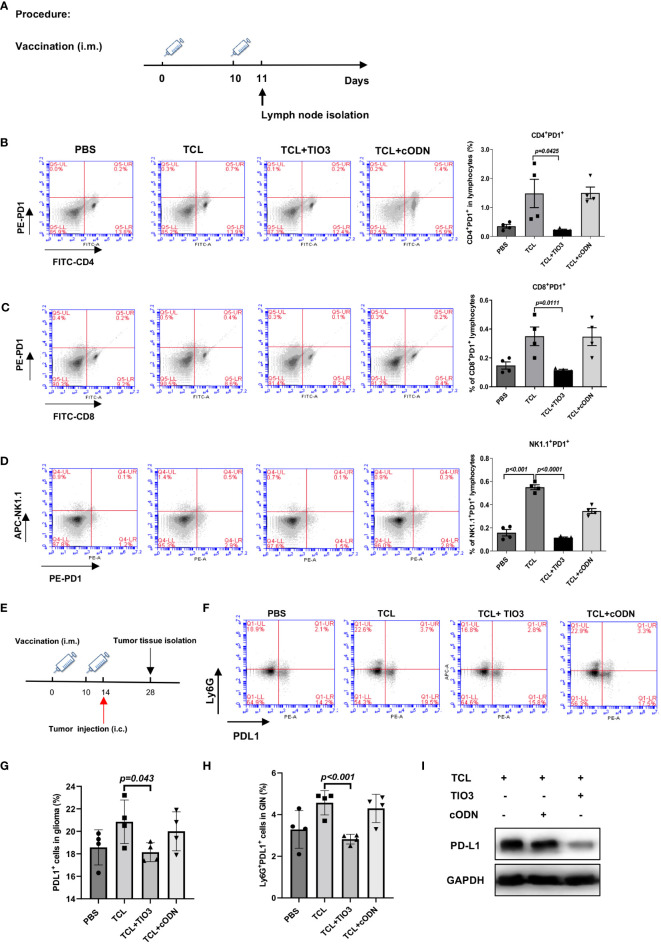
Inhibitory effects of TIO3 as glioma vaccine adjuvant on the expression of PD1 and PD-L1 on immune cells and tumor tissues in mice. **(A)** C57BL/6 mice (n = 4) were intramuscularly injected in the neck with PBS, TCL, TCL+TIO3 or TCL+cODN on day 0 and day 10, and 24h after the second immunization, the lymphocytes isolated from the mice were detected for the expression of PD1 on CD4^+^ T cells **(B)**, CD8^+^ T cells **(C)**, NK cells **(D)**. **(E)** The mice (n=4) were intramuscularly immunized with PBS, TCL, TCL+TIO3 and TCL + cODN in the neck on day 0 and 10, and then and then challenged i.c. with 2×10^4^ GL261 cells on day 14, 4 mice were sacrificed on day 28. **(F-H)** Percentage of PDL1^+^ cells in glioma tissue and glioma infiltrating neutrophils (Ly6G^+^) (GIN). **(I)** PD-L1 expression in glioma tissue isolated from the mice on day 28 by western blotting.

PD1 is an inhibitory receptor that is expressed by immune effector cells, including T cells, B cells and NK cells. To explore whether TCL+TIO3 could affect PD1 expression in immune cells, C57BL/6 mice were immunized with PBS, TCL, TCL+TIO3 or TCL+cODN on Day 0 and Day 10, and 24 h after the second immunization, splenocytes isolated from the mice were assessed for the expression of PD1 on CD4^+^ T, CD8^+^ T and NK cells by flow cytometry analysis (**Figure 6A**). Our results showed that TCL+TIO3 significantly inhibited PD1 expression in the CD4^+^ T, CD8^+^ T and NK cells compared with TCL alone (**Figures 6B-D**).

Since the high expression of PD-L1 in tumor tissues or neutrophils enables the suppression of T-cell function (24, 26), we determined whether TIO3 could impact the expression of PD-L1 in tumor tissues and tumor-infiltrating neutrophils. Mice were immunized with TCL, TCL+TIO3, TCL+cODN or PBS twice and then challenged with GL261 cells. On Day 14 after glioma inoculation, glioma tissues and tumor-infiltrating neutrophils were isolated to assess PD-L1 expression by western blotting and flow cytometry, respectively (**Figure 6E**). The results demonstrated that TIO3 significantly decreased PD-L1 expression and the percentage of Ly6G^+^ PD-L1^+^ neutrophils in glioma tissues (**Figures 6F-I**). These data indicated that TIO3 was capable of downregulating PD1-PDL1 inhibitory signaling in multiple immune cells, resulting in more potent activation of antitumor immunity.

### 3.7 Protective effect of TCL+TIO3 against murine glioma on *in situ* therapeutic model mice

**Figure 7:**
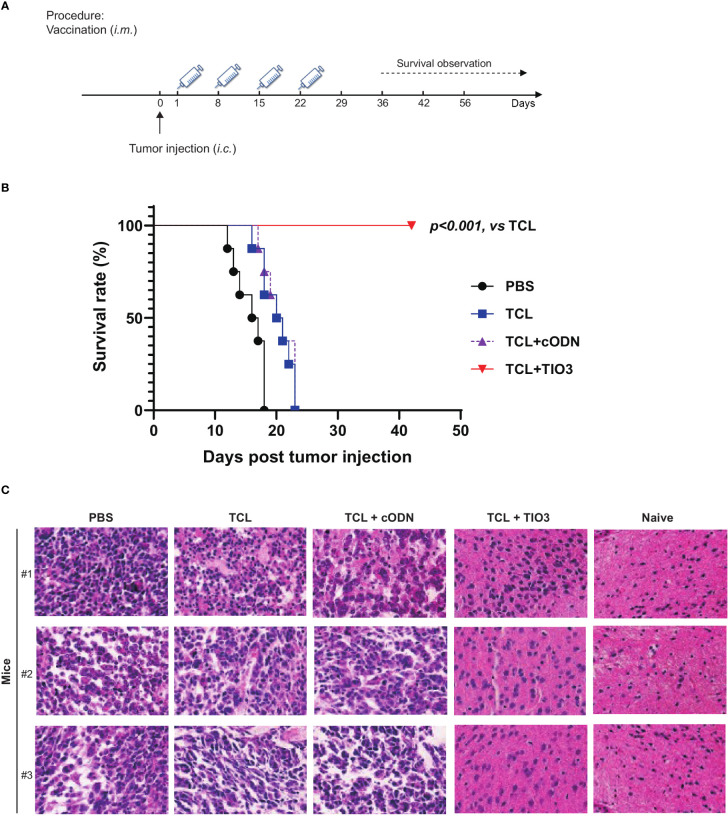
Therapeutic protective effect of TIO3 as vaccine adjuvant on the *in-situ* glioma model in mice. TIO3 as a glioma vaccine molecular adjuvant combined with GL261 TCL to treat GL261-bearing mice. **(A)** The experimental procedure: C57BL/6 mice (n=8) were intracranial (i.c.) inoculated with 2×10^4^ GL261 cells in right caudate nucleus of the brains on day 0, and then were i.m. immunized in the neck with PBS, TCL, TCL+TIO3 or TCL+cODN on day 1, 8, 15 and day 22. **(B)** The survival of GL261-bearing mice. **(C)** Brain tissues were taken for histopathological examination with HE staining analysis on the 18th day. The three mice received PBS died within 18 days after the GL261 cell inoculation, and their brain samples were analyzed immediately after death. (Magnification, ×400).

To explore whether the glioma vaccine TCL+TIO3 could prolong the survival of mice with glioma in an *in situ* therapeutic model, mice were injected with GL261 cells on Day 0 and then immunized four times with TCL, TCL+TIO3, TCL+cODN or PBS on Days 1, 8, 15 and 22. Interestingly, the survival results showed that TCL+TIO3 significantly prolonged the survival of mice bearing glioma, and no mice died within 40 days (**Figures 7A, B**), while all mice in the other groups died within 23 days. These data indicated that TCL+TIO3 also induced a significant protective effect against murine glioma in an *in situ* therapeutic model.

Furthermore, brain tissues were taken for histopathological examination on the 18th day post-injection. Although glioma cells could be found in the mice of each group, the amount of tumor cells within the isolated tissue area of TCL+TIO3 mice was less than that of the PBS, TCL and TCL+cODN groups. The microscopy results demonstrated that the glioma tissue in those groups showed enlarged intercellular spaces, irregular arrangement, and uneven arrangement of intercellular substances. Moreover, in the TCL+TIO3 group, increased infiltration of immune cells appeared in the brain tumor tissue, which may be associated with the enhancement of anti-glioma immunity (**Figure 7C**). The above results suggested that TCL+TIO3 also induced massive immune cell infiltration into brain tissues to enhance antitumor immunity, which facilitated limiting the growth of tumors and prolonging the survival of glioma-bearing mice.

## 4 Discussion

TGF-β2 inhibitors, including monoclonal antibodies, small molecules, and antisense oligonucleotides, have been used alone to treat tumors. However, little is known about their use as tumor vaccine adjuvants. In our previous study, a self-designed TGF-β2 inhibitory oligodeoxynucleotide, TIO3, was used as an adjuvant that was formulated with multiple microbial vaccines to induce strong and persistent antibody responses (12). Therefore, it is worthwhile to investigate whether TIO3 can also be used as a tumor vaccine adjuvant. In this study, we found that TIO3 is capable of being used as an effective vaccine adjuvant coupled with glioma TCL (TCL+TIO3) to induce potent antitumor immune responses and prolong the survival of glioma-bearing mice in prophylactic and therapeutic *in situ* models. In particular, TCL+TIO3 not only induced the activation of CD4^+^ T, CD8^+^ T and NK cells but also inhibited PD1 and PD-L1 expression in multiple immune cells and glioma tissues by suppressing the expression of TGF-β2.

TIO3 could significantly inhibit the expression of TGF-β2 in various cells. For example, TIO3 formulated in microbial vaccines dramatically reduced surface-bound TGF-β2 expression on CD19^+^ B cells in splenocytes, resulting in an elevation of IgG levels (12). In this study, we demonstrated that TIO3 also broadly inhibits sTGF-β2 expression in tumor cell lines and glioma tissues, as well as in antitumor effector CD4^+^ and CD8^+^ T cells. TIO3 has also been confirmed capable of entering lung tumor tissues and tumor cells *in vitro* and *in vivo* (27). Our previous study revealed that the percentages of Cy3^+^ cells reached 100% of the cultured LLC cells at 24 h after Cy3-TIO3 addition, and 50% of tumor tissue cells from tumor-bearing mice at 24 h post i.p. injection of Cy3-TIO3. Thus, we think the actual targets of TIO3 are T cells and tumor cells in tumor immunotherapy. As reported, TGF-β2 can negatively regulate the expression of HLA-DR antigen on the surface of human malignant glioma cells. Moreover, TGF-β2 is sufficient to downregulate the surface expression of MHC molecules and may enhance the ability of tumor cells to evade immune responses (28). Thus, TGF-β2 has also been named glioblastoma-derived T-cell suppressor factor, a central molecule maintaining the malignant phenotype of glioblastoma (29). As expected, TIO3 significantly upregulated the mRNA and protein expression of MHC I molecules in GL261 and U251 cells. The upregulation of MHC-I molecules that present tumor antigens could promote the lysis of tumor cells by cytotoxic T lymphocytes. In addition, TIO3 also upregulated the expression of CD40, CD80, CD86, and MHC II molecules on CD11c^+^ dendritic cells (12), facilitating antigen presentation to induce cytotoxic effects against microbes and tumors.

TIO3 is a short, single-stranded ODN, usually, ODNs enter cells *via* endocytosis (30). Also, it could be a ligand for TLR9. So, we determined whether TIO3 could activate TLR9 signals. In our previous study (27), there were no changes of TLR9 expression between the TIO3 and PBS groups. Moreover, the TIO3 didn’t affect the mRNA expression of the expression of downstream molecules of TLR9 signals, such as the IRF5, TNF-α an IFN-α. Thus, we think TIO3 doesn’t bind to TLR9 and stimulate innate immune response. Noticeably, the TIO3 was specially designed for the treatment of gliomas, and it was confirmed that it specifically inhibited the mRNA expression of TGF-β2 but not the expression of TGF-β1 and TGF-β3 (12). This is because the TIO3 complementary sequence is 100% identical to the original mRNA sequence of TGF-β2, while the mRNA sequence of TGF-β1 and TGF-β3 just have 63% and 57.89% similarity, respectively. TGF-β2 triggers signaling in cells *via* binding to a TGF-β receptor complex composed of two type I TGF-β receptors and two type II TGF-β receptors. Then, these receptors recruit and phosphorylate receptor regulated Smad2, Smad3 and other Smads to forms a heterodimeric complex. This complex then translocates into the cell nucleus where it binds with nuclear co-factors to regulate the transcription of various target genes involved the cancer progression [7]. Our result showed that TIO3 reduced the phosphorylation level of Smad3 in GL261 cells, compared to the PBS and cODN (**Figure S3**). This result is consistent with the results of TGF-β2 inhibition and tumor recession.

T cells and NK cells are both cytotoxic effector cells of the immune system against tumors. We found that TCL+TIO3 induced the activation of CD8^+^ T cells, which resulted in the secretion of IFN-γ, which can kill tumor cells directly. In addition to the cytokine effect, CD8^+^ T cells can release cytotoxic granules and activate the Fas/FasL pathway, resulting in tumor cell apoptosis (31). Moreover, the cytotoxic effect confirmed that TCL+TIO3 induced significant specific CTLs in glioma-bearing mice. Indeed, specific CD4^+^ T-cell responses are also closely related to the tumor-killing effect (31). In particular, most tumor neoantigens are recognized by CD4^+^ T cells and not CD8^+^ T cells (32). The reason may be that MHC class II molecules are not constitutively expressed in most tumor cells, and therefore it is necessary for DCs to present tumor neoantigens and activate tumor-specific CD4^+^ T cells. Thus, tumor vaccines prepared with new epitopes of CD4^+^ T cells can effectively control the development of advanced tumors. TCL contains many neoantigens, and immunization of mice with new epitopes restricted by immunodominant MHC-II molecules can trigger strong tumor-specific CD4^+^ T-cell and CD8^+^ T-cell responses (33, 34). Our data also confirmed that TIO3 exerts an antiglioma effect by promoting the activation of CD4^+^ T cells. In addition, NK cells play an important role in innate defenses against glioma growth. NK cells not only kill tumor cells but also induce tumor cells to change the expression of HLA-I, PD-L1, or NKG2D-L and modulate their susceptibility to the immune response (35). In this study, TCL+TIO3 induced an increased population of NK cells and higher ratios of CD69^+^ or NKG2D^+^ NK cells in splenocytes compared with those of the TCL group, resulting in stronger cytotoxic activities against glioma.

In this study, a massive number of glioma-specific lymphocytes were induced in the periphery by TCL+TIO3. Generally, lymphocytes are recruited from the blood circulation to the tumor site and infiltrate the tumor mass, such as melanoma and colorectal and ovarian carcinoma. Tumor-infiltrating lymphocytes (TILs) are considered to be important antitumor effector cells, and the accumulation of activated lymphocytes at tumor sites is considered a good indicator of the regulation of tumor development and growth. As reported, TILs are mainly composed of T cells, B cells, and NK cells (36). However, it is difficult for lymphocytes to infiltrate glioma tissues through the blood−brain barrier (BBB). The presence of the BBB strictly restricts the movement of immune cells and soluble mediators from the periphery to the central nervous system. However, TILs have been detected in glioma patients (37). Glioma is a very aggressive tumor that is characterized by extensive proliferation and migration. Uncontrolled and fast growth of glioma can lead to the disruption of chimeric and fragile vessels, resulting in damage to the BBB. Therefore, lymphocytes can enter the brain through the damaged BBB to contain and kill glioma cells. Additionally, lymphocytes can enter the perivascular or mesenteric space from the endothelial cell wall of the blood−brain barrier *via* multiple adhesion molecules and signaling molecules and then cross the boundary membrane of glial cells and enter the central nervous system (38). Our histopathological results showed increased TILs in brain glioma tissues in the TCL+TIO3 group, which may be associated with the enhancement of antiglioma immunity. In line with these results, glioma patients after surgery were administered an autologous glioma TCL extracranially, which induced the massive infiltration of T lymphocytes into the brain tissues from the periphery, facilitating the clearance of the remaining glioma cells and preventing the recurrence of gliomas (34).

In addition to the activation of antitumor effector cells, TIO3 also reduced the expression of PD1 on CD4^+^ T cells, CD8^+^ T cells and NK cells and inhibited PD-L1 expression in glioma tissues and tumor-infiltrating neutrophils. Immune checkpoint signals such as PD1 and PD-L1 are key modulators of the antitumor T-cell immune response. In a tumor state, the interaction of PD-L1 on tumor cells with PD1 on T cells can inhibit the cytotoxic ability of T cells against tumor cells (39, and PD-L1 expression on neutrophils also enables the suppression of T-cell function. Moreover, TGF-β promotes PD1-PDL1 signaling in tumor immune escape. TGF-β can upregulate the expression of PD1 and CTLA-4 on T lymphocytes and attenuate the cytotoxicity of T lymphocytes toward tumor cells *in vitro* and *in vivo* (40). Additionally, TGF-β present in the tumor microenvironment orchestrates tumor cell expression of the PD-L1 molecule, and therefore the PD-L1 level is highly correlated with the tumor TGF-β level (41). Thus, immunotherapy with inhibitors of PD1 or PD-L1 appears to prevent the tumor from evading the immune system in this way (39). Similarly, switching to a new strategy that inhibits TGF-β2 expression could reduce PD1 and PD-L1 production. As shown by the results of this study, TIO3 had a synergistic effect on the inhibition of PD1 expression in immune cells and PD-L1 expression in glioma cells by interfering with the expression of TGF-β2, resulting in more potent activation of antitumor immunity.

Overall, TIO3 as a vaccine adjuvant activated antitumor effector T cells and NK cells by inhibiting the immunosuppressive cytokine TGF-β2 and PD1 and PD-L1 expression on immune cells and tumor cells. Importantly, TIO3 formulated with TCL effectively prolonged the survival of glioma-bearing mice in both *in situ* prophylactic and therapeutic models. Interestingly, we found that TIO3 also had a monotherapeutic effect on the xenograft and *in situ* therapeutic mouse models of glioma (**Figures S4**, **S5**), but the survival dates of mice were shorter than those with TCL+TIO3 immunization. This result suggests that TIO3 formulated with glioma cell lysate as a new type of glioma vaccine is a more promising immunotherapy strategy against glioma. Particularly, the preventive strategy of TCL+TIO3 in tumor immunotherapy induced a strong tumor-specific immunologic memory. In fact, the preventive tumor immunotherapy at early stage of tumor development or for preventing tumor recurrence could induce the formation of a tumor microenvironment (TME), which is conducive to the DC activation as well as the recruitment and activation of the effector immune cells, particularly CD8^+^ T cells and NK cells (27). Therefore, it has great potential application value for immunizing patients with TIO3 formulated with autologous TCL derived from excised glioma tissues, with the expectation of preventing the recurrence of glioma after surgery.

## Data availability statement

The original contributions presented in the study are included in the article/**Supplementary Material**. Further inquiries can be directed to the corresponding authors.

## Ethics statement

The animal study was reviewed and approved by The ethics committee of the College of Basic Medical Sciences of Jilin University.

## Author contributions

MY and YLY contributed to the conception and design of the study and wrote the manuscript. LT, XS, LY performed experiments, analyzed data, and performed the statistical analysis. ZW, YPY, LW, XZ and PZ contributed to manuscript revision, read and approved the submitted version. All authors contributed to the article and approved the submitted version.

## Supplementary material

Supplementary Figure 1 The TIO3 distribution in B cells. The lymphocytes and spleen cells from naive mice (n = 4) were incubated with green-fluorescent Alexa-488-TIO3 or Alexa-488-cODN for 24 h, then stained with red-fluorescent (PE-labeled) mAbs against CD19, respectively, followed by counterstaining with DAPI, a blue fluorescent DNA dye for staining the nucleus. The resultant cells were observed under the confocal microscope (Scale bar represents 50 µm).

Supplementary Figure 2 The IFN-γ production of the cultured lymphocyte from the mice immunized with TCL+TIO3. The lymphocytes were isolated from draining lymph nodes of mice in PBS, TCL, TCL+TIO3 or TCL+cODN groups, and co-cultured with GL261 cells *in vitro*. 48h later, we collected the supernatant from the medium and detected the expression of IFN-γ by ELISA.

Supplementary Figure 3 Western blot analysis of the expression of p-Smad3 in each group. GL261 cells was cultured with PBS, TIO3 (10 μg/mL) or cODN (control ODN) for 24 h, respectively, and the phosphorylation of SMAD3 protein was measured by western blot.

Supplementary Figure 4 Protective effect of TIO3 as a therapeutic agent on the xenograft therapeutic mouse model. We subcutaneously inoculated mice with 5×10^5 GL261 cells, then PBS or TCL+TIO3 were intraperitoneally immunized four times with TIO3 or PBS on Days 1, 8, 15 and 22. Tumor growth **(A)** and survival **(B)** of the mice were monitored throughout the procedure.

Supplementary Figure 5 Protective effect of TIO3 as a therapeutic agent on the *in situ* therapeutic mouse model. **(A)** The experimental procedure: C57BL/6 mice (n =6) were intracranially (i.c.) inoculated with 2×10^4^ GL261 cells on day 0, and then were intraperitoneal injection (i.p.) with TIO3 or PBS on day 1, day 3, day 5 and day 7. **(B)** The survival rate of GL261-bearing mice was recorded.
