# Supplementary figures and images for: Incorporation of a TGF-β2-inhibiting oligodeoxynucleotide molecular adjuvant into a tumor cell lysate vaccine to enhance antiglioma immunity in mice
Source: Front Immunol. 2023 Jan 27;14:1013342. doi: 10.3389/fimmu.2023.1013342 (PMC9914600; doi:10.3389/fimmu.2023.1013342)

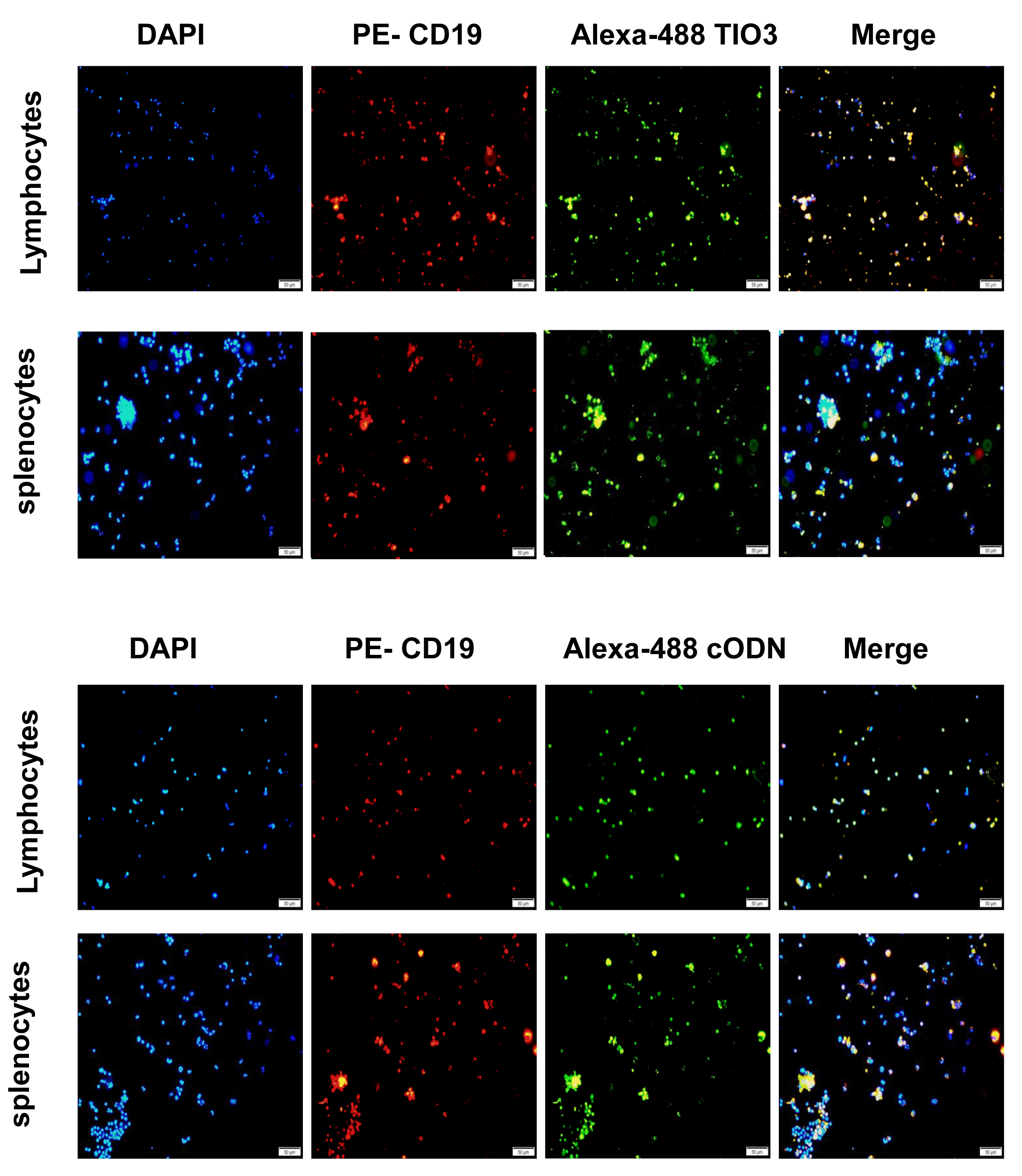

Supplement: Supplementary Figure 1 — The TIO3 distribution in B cells. The lymphocytes and spleen cells from naive mice (n = 4) were incubated with green-fluorescent Alexa-488-TIO3 or Alexa-488-cODN for 24 h, then stained with red-fluorescent (PE-labeled) mAbs against CD19, respectively, followed by counterstaining with DAPI, a blue fluorescent DNA dye for staining the nucleus. The resultant cells were observed under the confocal microscope (Scale bar represents 50 µm). [file Image_1.tif]

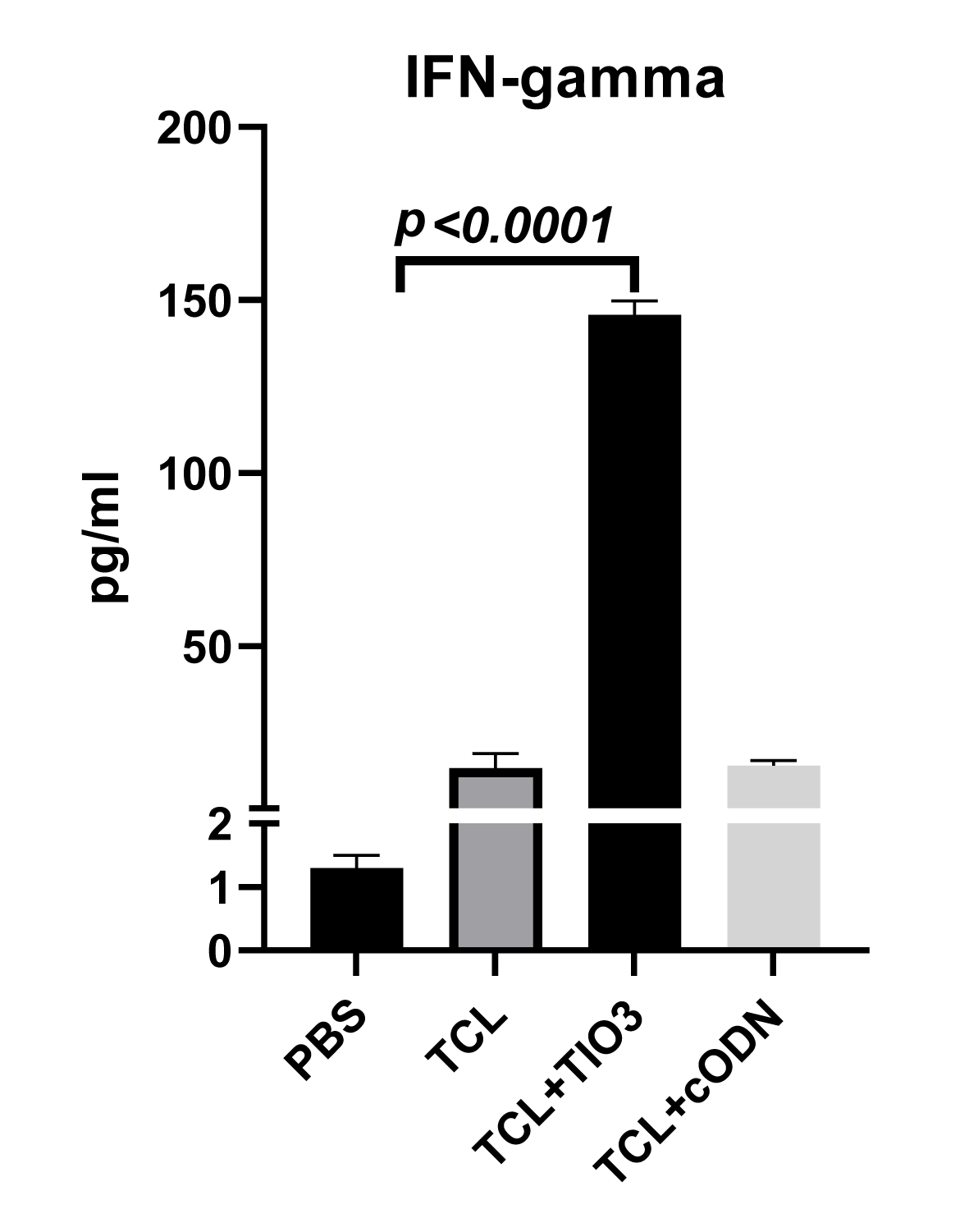

Supplement: Supplementary Figure 2 — The IFN-γ production of the cultured lymphocyte from the mice immunized with TCL+TIO3. The lymphocytes were isolated from draining lymph nodes of mice in PBS, TCL, TCL+TIO3 or TCL+cODN groups, and co-cultured with GL261 cells in vitro. 48h later, we collected the supernatant from the medium and detected the expression of IFN-γ by ELISA. [file Image_2.tif]

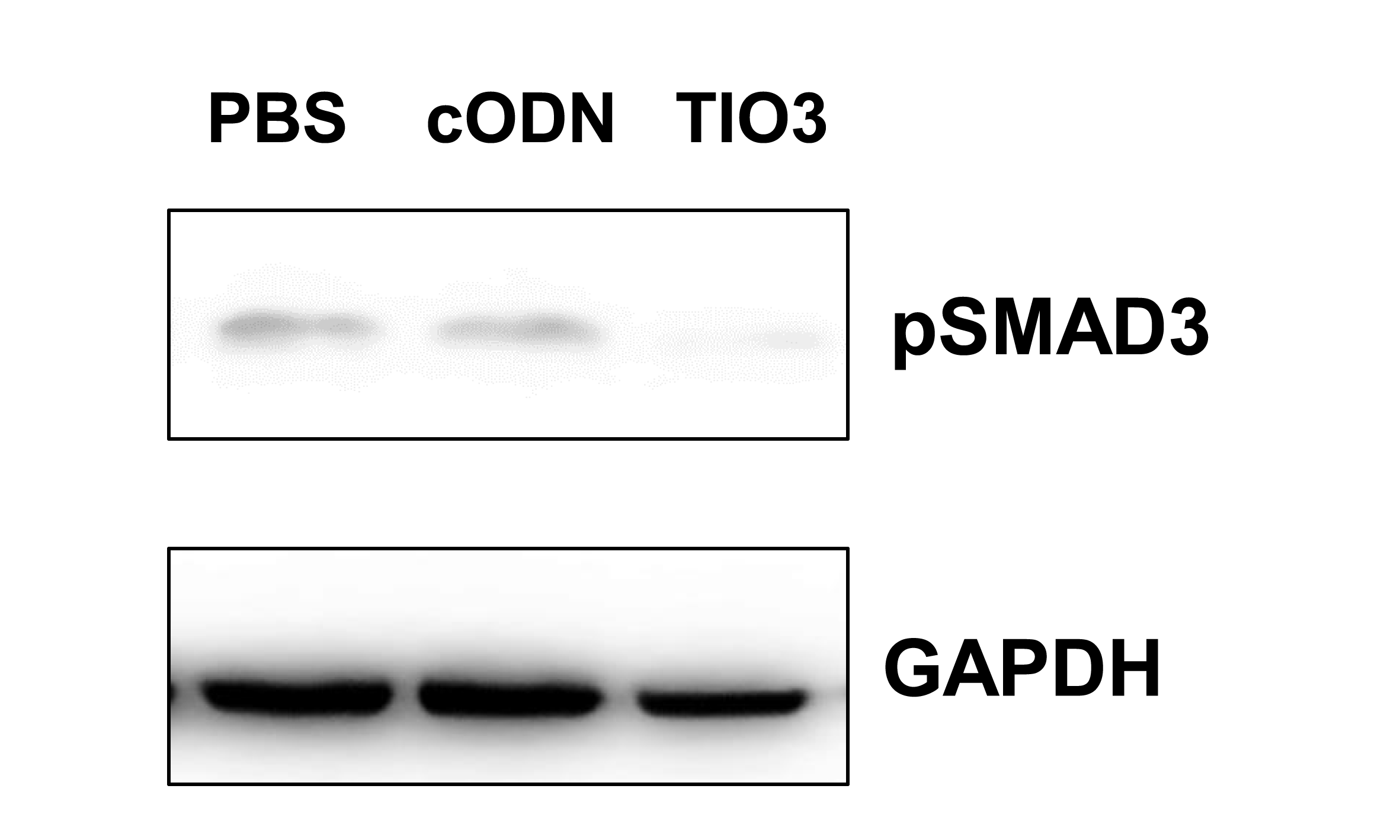

Supplement: Supplementary Figure 3 — Western blot analysis of the expression of p-Smad3 in each group. GL261 cells was cultured with PBS, TIO3 (10 μg/mL) or cODN (control ODN) for 24 h, respectively, and the phosphorylation of SMAD3 protein was measured by western blot. [file Image_3.tif]

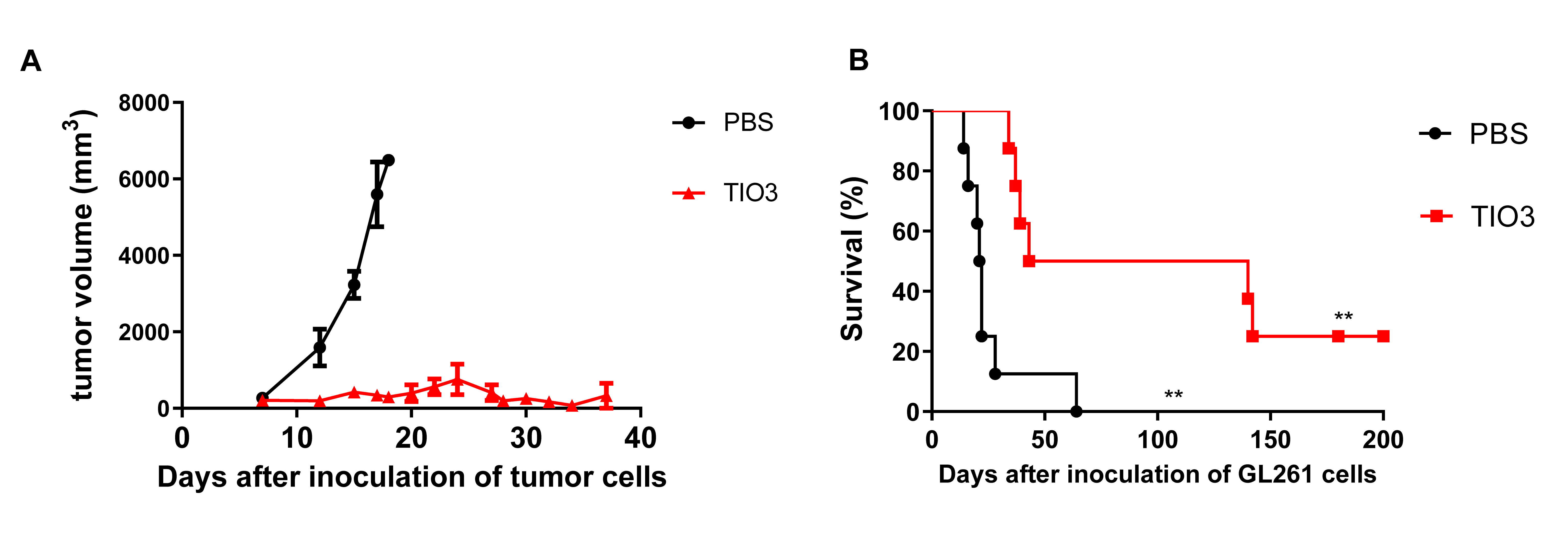

Supplement: Supplementary Figure 4 — Protective effect of TIO3 as a therapeutic agent on the xenograft therapeutic mouse model. We subcutaneously inoculated mice with 5×10^5 GL261 cells, then PBS or TCL+TIO3 were intraperitoneally immunized four times with TIO3 or PBS on Days 1, 8, 15 and 22. Tumor growth (A) and survival (B) of the mice were monitored throughout the procedure. [file Image_4.tif]

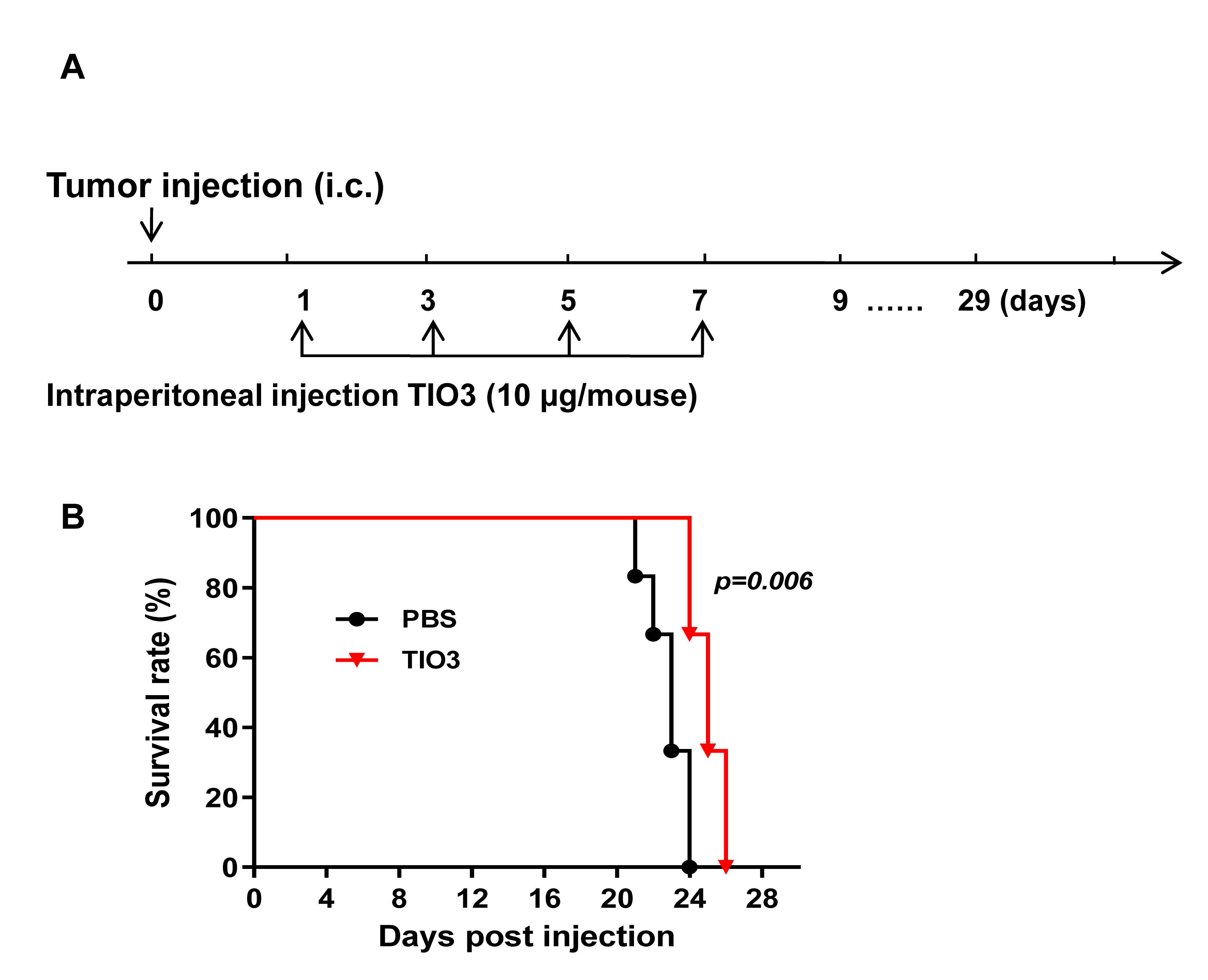

Supplement: Supplementary Figure 5 — Protective effect of TIO3 as a therapeutic agent on the in situ therapeutic mouse model. (A) The experimental procedure: C57BL/6 mice (n =6) were intracranially (i.c.) inoculated with 2×10^4^ GL261 cells on day 0, and then were intraperitoneal injection (i.p.) with TIO3 or PBS on day 1, day 3, day 5 and day 7. (B) The survival rate of GL261-bearing mice was recorded. [file Image_5.tif]
